# *Candida albicans* Uses the Surface Protein Gpm1 to Attach to Human Endothelial Cells and to Keratinocytes via the Adhesive Protein Vitronectin

**DOI:** 10.1371/journal.pone.0090796

**Published:** 2014-03-13

**Authors:** Crisanto M. Lopez, Reinhard Wallich, Kristian Riesbeck, Christine Skerka, Peter F. Zipfel

**Affiliations:** 1 Department of Infection Biology, Leibniz Institute for Natural Product Research and Infection Biology (Hans Knöll Institute), Jena, Germany; 2 Institute of Immunology, University of Heidelberg, Heidelberg, Germany; 3 Medical Microbiology, Department of Laboratory Medicine Malmö, Lund University, Malmö, Sweden; 4 Friedrich Schiller University, Jena, Germany; University of Kentucky College of Medicine, United States of America

## Abstract

*Candida albicans* is a major cause of invasive fungal infections worldwide. Upon infection and when in contact with human plasma as well as body fluids the fungus is challenged by the activated complement system a central part of the human innate immune response. *C. albicans* controls and evades host complement attack by binding several human complement regulators like Factor H, Factor H-like protein 1 and C4BP to the surface. Gpm1 (Phosphoglycerate mutase 1) is one fungal Factor H/FHL1 -binding protein. As Gpm1 is surface exposed, we asked whether Gpm1 also contributes to host cell attachment. Here, we show by flow cytometry and by laser scanning microscopy that candida Gpm1 binds to human umbilical vein endothelial cells (HUVEC) to keratinocytes (HaCaT), and also to monocytic U937 cells. Wild type candida did bind, but the candida *gpm1Δ/Δ* knock-out mutant did not bind to these human cells. In addition Gpm1when attached to latex beads also conferred attachment to human endothelial cells. When analyzing Gpm1-binding to a panel of extracellular matrix proteins, the human glycoprotein vitronectin was identified as a new Gpm1 ligand. Vitronectin is a component of the extracellular matrix and also a regulator of the terminal complement pathway. Vitronectin is present on the surface of HUVEC and keratinocytes and acts as a surface ligand for fungal Gpm1. Gpm1 and vitronectin colocalize on the surface of HUVEC and HaCaT as revealed by laser scanning microscopy. The Gpm1 vitronectin interaction is inhibited by heparin and the interaction is also ionic strength dependent. Taken together, Gpm1 the candida surface protein binds to vitronectin and mediates fungal adhesion to human endothelial cells. Thus fungal Gpm1 and human vitronectin represent a new set of proteins that are relevant for fungal attachment to human cells interaction. Blockade of the Gpm1 vitronectin interaction might provide a new target for therapy.

## Introduction

The human opportunistic pathogen *Candida albicans* is the leading cause of fungal diseases worldwide [1]. *C. albicans* causes systemic and also mucocutaneous infections which are frequent in immunocompromised individuals [2]. Upon infection, *C. albicans* is challenged by host innate immune reactions and the fungal pathogen employs several strategies to evade host immune response, to cross tissue barriers and to gain access to different tissue layers. *C. albicans* evades the human innate immune system and controls complement attack by binding human complement plasma regulators, such as Factor H, Factor H-like 1 protein (FHL-1), CFHR1 and C4BP [3], [4], [5]. Bound to the fungal surface, these regulators block complement cascade at various levels, inhibit cascade progression and assist in the degradation of the opsonin C3b [6], [7], [8]. Thereby protecting *C. albicans* from the damaging effects of the activated complement system and form opsonophagocytosis. At present five candida proteins are identified which bind human complement- and immune regulators [6], 7 *i.e.*, Pra1 (pH-regulated antigen 1) [9], [10], Gpd2 (glycerol-3-phosphate dehydrogenase 2 [11], Hgt1 (high-affinity glucose transporter 1) [12] and Gpm1 (phosphoglycerate mutase) [13]. Gpm1 was the first fungal surface protein identified, that binds the complement regulators Factor H, FHL-1 and plasminogen [13], [14]. As a cytoplasmic protein Gpm1 catalyzes glycolysis and glyconeogenesis. Gpm1-bound Factor H and FHL-1 are functionally active and act as cofactors for Factor I mediated degradation of C3b. In addition, Gpm1 binds plasminogen, and plasminogen bound to GPM1 is accessible for the activator uPa, and activated plasmin degrades host extracellular matrix (ECM) proteins and C3b [13]. Tthus Gpm1 bound plasminogen contributes to immune and tissue evasion [15].

*C. albicans* adheres to human endothelial cells [16], keratinocytes [17], [18], oral epithelial cells [19], to subendothelial matrix [20], and gain access into host cells and ultimately into deeper tissue layers. The fungus uses integrin-like receptors [21], glycans, mannnoproteins [22], phospholipidomannan [23] and other cell wall-associated proteins to contact different human cell receptors and components of the ECM [24], [25].

Vitronectin is a multifunctional human adhesion protein, is part of the extracellular matrix, is present in plasma and is a complement regulator [26], [27]. Vitronectin is a 75 kDa human serum protein and a component of the ECM. This adhesive glycoprotein binds to heparin and to the human integrin receptors α_v_β_3_ and α_v_β_5_ [28], [29]. Vitronectin aids in cell proliferation, adhesion and angiogenesis [30], [31]. In addition, vitronectin is a regulator of the terminal complement pathway [32]. Based on these multiple functions many pathogenic microbes bind human vitronectin to their surface [33]. Surface attached vitronectin is used for immune evasion, for ECM adherence, adhesion to human cells and subsequent tissue invasion (reviewed in [33]. Many pathogenic bacteria bind human vitronectin to their surface and use surface-attached vitronectin to bind to human cells and to ECM components. Apparently many pathogenic microbes including Gram negative bacteria but also Gram positive bacteria and fungi control the action of TCC. [7], [34], [35]. The Gram-positive pathogenic bacterium *S. pneumoniae* recruits human vitronectin to its surface and bound vitronectin aids in cell contact, ECM interaction and tissue invasion [36]. Pathogenic microbes bind human vitronectin to their surface include *Streptococcus pneumoniae*, *Pseudomonas aeruginosa*, *Neisseria meningitidis*, *N. gonorrhoeae*, as well as *Moraxella catarrhalis*, *Haemophilus influenza*, *Neisseria meningitidis*, *Streptococcus pneumoniae*, *S. pyogenes* and *Staphylococcus aureus* [37], [38], [39], [40], reviewed in [33]. Several surface proteins of these pathogenic microbes that bind vitronectin have been identified. These include protein E from NT *H influenza*, UspA2 from *M. catarrhalis*, Opc by N. *meningitidis*, OpA by *N. gonorrhoeae* and PspC by *S. pneumonia* [38], [39], [40], [41], [42], [43]. *S. pneumonia* similar to other pathogenic microbes binds vitronectin via the heparin-binding region, leaving the N-terminal integrin-binding site exposed and free for interaction with cell surface receptors and other ligands [34], [40]. Many of these microbial proteins bind vitronectin via the heparin-binding domains [33].

The fungal pathogen *C. albicans* binds vitronectin, both as a soluble plasma protein or as a component of the ECM. Vitronectin bound to the fungal surface seems relevant for the contact of fungi with human cells [44], [45], [46].

Here we show that Gpm1, the candida surface protein and moonlighting protein binds human vitronectin and Gpm1 mediates fungal binding and attachment to human endothelial cells (HUVEC), to keratinocytes and to monocytic U937 cells. Gpm1 is central and relevant for fungal contact with HUVECs, as the *Candida gpm1* knock-out mutant bound with lower intensity to these human endothelial cells. In addition, GPM1 when conjugated to the surface of latex beads mediates attachment and contact to these human cells. In addition we identify vitronectin as a new ligand for candida Gpm1, and Gpm1 by binding to vitronectin on the surface of endotheilal cells and keratinocytes mediates contact to human cells and likely the extracellular matrix.

## Materials and Methods

### *C. albicans* strains and growth conditions

*C. albicans* SC5314 was cultivated in YPD broth [2% (w/v) glucose, 2% peptone, 1% yeast extract]. *C. albicans GPM1* mutant strains (*gpm1Δ/Δ*, *gpm1Δ*, and *gpm1Δ/Δ::GPM1*) [13] were cultivated in Saboraud Glycerol (SG) broth [3% (w/v) glycerol, 2% peptone from casein, 1% yeast extract] at 180 rpm at 30°C prior to experiments. Fungal cultures were maintained on YPD or SG agar plates at 4°C and colonies were transferred to new agar plates every two weeks. Agar media were prepared as above with addition of 1.5% (w/v) agar.

### Maintenance and cultivation of human cell lines

Human umbilical vein endothelial cells (HUVEC) and human keratinocytes (HaCaT) were purchased from American Type Culture Collection (Manassas, VA). HUVEC and HaCaT were maintained in DMEM and U937 cells were cultivated in RPMI 1640, (Lonza). Media were supplemented with 10% fetal calf serum (FCS) (PAA Laboratories, Austria), 1% ultraglutamine 1 (Lonza), and 0.055% gentamicin sulfate (Lonza). Human cells were incubated at 37°C with 5% CO_2_ and passaged every three days. Cells with less than 30 passages were used in the experiments.

### Recombinant Gpm1

Gpm1 was expressed and purified as described [13]. In brief, Gpm1 was recombinantly expressed as a His-tagged protein in *Pichia pastoris* strain X33. Protein expression was induced with 1% methanol. The culture supernatant was harvested after 3 days of expression, and recombinant Gpm1 was purified by nickel affinity chromatography using HisTrap column (GE Healthcare) in an Äkta fast protein liquid chromatography system (GE Healthcare). Elute fractions were concentrated in PBS using Vivaspin 15R concentrators with a cut off of 10 kDa (Vivascience, Hannover, Germany).

### Biotinylation of proteins

Biotinylated Gpm1 and BSA were generated using the EZ-Link Sulfo-NHS Biotinylation kit (Pierce Biotechnology, IL) by following the manufacturer's instructions. Briefly, 200 µl of protein solution (1 mg/ml) was added with appropriate volume of 10 mM Sulfo-NHS-biotin solution to achieve a 20-fold molar excess of biotin reagent. Then the reaction mixture was incubated for 60 min at RT. To exchange the buffer to DPBS and remove excess biotin, the reaction mixture was centrifuged in Zeba Spin Desalting Column (Pierce, IL). The pure biotinylated protein was stored in DPBS at −20°C until use.

### Gpm1- or BSA-coated latex beads

Latex beads were coated with recombinant Gpm1 or biotinylated BSA by a modified method of Phan *et al.* [11]. Briefly, fluorescent blue amine–modified polystyrene latex beads (2.0 µm mean particle size; Sigma-Aldrich) were washed first with DPBS and then with coupling buffer (0.2 M Na_2_HCO_3_ [pH 8.5] and 0.5 M NaCl). Thereafter, beads were incubated with Gpm1 (0.5 mg/ml) or coupling buffer at 37°C for 30 min. Rabbit serum (1%) was added to the beads that had been coated with Gpm1, while biotinylated BSA (1 mg/ml) was added to control beads. Beads were incubated at 37°C for 30 min, sonicated briefly and blocked by addition of unlabeled BSA (10 mg/ml in coupling buffer) and finally incubated for 1 h. After washing twice with DPBS containing 10 mg BSA/ml, beads were suspended in DPBS containing BSA (2 mg/ml). Binding of Gpm1 to the beads was verified by immunofluorescence using a monoclonal antibody against Gpm1.

### Gpm1 binding to human cells

Determination of the binding of recombinant Gpm1 to both human HUVEC and kertinocytes was done using flow cytometry and laser scanning microscopy (LSM). For the flow cytometry experiment, human cells were collected and washed twice with PBS supplemented with 1% BSA (washing buffer). Recombinant Gpm1 was added to the cells at varied concentrations (0, 1.25, 2.5 and 5.0 µg), and the mixture was incubated for 30 min at 37°C. After washing twice with washing buffer, bound Gpm1 was detected with rabbit polyclonal anti-Gpm1 antiserum (dilution 1∶100) followed by Alexa 647-labeled goat anti-rabbit IgG (1∶200) and analyzed by flow cytometry. To analyze whether vitronectin affects Gpm1-binding to HUVEC and keratinocytes, vitronectin (0.1 µg/µl) was pre-incubated with biotinylated Gpm1, and thereafter the mixture was added to HUVEC and keratinocytes. After washing, bound Gpm1 was quantified using streptavidin-conjugated Cy5 and flow cytometry. To visualize bound Gpm1 on human cells, HUVEC or keratinocytes were grown on 14-mm coverslip in a 24-well tissue culture plate until confluent. Cells were added with 10 ug/ml recombinant Gpm1 in FCS-free DMEM then incubated for 1 hour at 37°C and 5% CO_2_. After several washes, rabbit polyclonal Gpm1 antiserum was added (dilution 1∶100) and incubated for 30 min at RT. Following washing, Alexa 488-labeled goat anti-rabbit IgG (1∶200) was added to wells for 30 min at RT. Thereafter, coverslips were removed and the cells were analyzed using a laser scanning microscope.

### Measurement of host cell-associated C. albicans or latex beads

The number of *C. albicans* cells or latex beads which were associated (adhered and endocytosed) with HUVECs was followed by flow cytometry or by confocal laser scanning microscopy. HUVECs were grown in 24-well tissue culture plate until confluent. Prior to inoculation with *C. albicans* yeast cells, human cells were stained with the carbocyanine DiO and DiD (Molecular Probes). After several washes, DiD-stained fungal cells (1.0×10^5^) were inoculated and the plate was incubated for 2 h at 37°C with 5% CO_2_. HUVECs were removed from wells by using Accutase. Host cell-associated *C. albicans* were measured by determining the number of host cells with double positive signal for DiO and DiD after gating using the forward and sideward scatters. To view and measure the amount of Gpm1-coated fluorescent latex beads which became associated with HUVECs, indirect fluorescence was used. Gpm1- or biotinylated BSA-coated beads (2.5×10^5^) were added to confluent HUVECs on 14-mm diameter coverslips in 24-well tissue culture plates for 45 min at 37°C with 5% CO_2_. After several washings, HUVECs on coverslip were analyzed with 100× magnification under the oil immersion objective using a confocal laser scanning microscope. Each coverslip was divided into four quadrants where a 5×5 tile scan was done per quadrant. The mean intensity of the blue fluorescence per tile scan was obtained using the ZEN 2009 software.

### Binding of extracellular matrix (ECM) proteins to Gpm1

Binding of different human extracellular matrix (ECM) proteins to Gpm1 was analyzed by enzyme-linked immunosorbent assay (ELISA). Gpm1 or BSA (0.25 µg) in carbonate-bicarbonate buffer was immobilized on a 96-well microtiter plate overnight at 4°C. After washing one time with distilled deionized water and one time with the washing buffer (PBS+0.05% Tween 20), the plate was blocked with the blocking solution (4% milk powder+2% bovine serum albumin in PBS) for 2 h at room temperature (RT). After washing twice with washing buffer, 0.75 µg of ECM protein (fibronectin, Vitronectin, laminin, fibrinogen, collagen I, collagen III or collagen IV, Sigma) was added to the plate and incubated for 1 h at RT. After washing twice with washing buffer, the corresponding primary antibody (dilution 1∶1,000 in blocking solution) was added to the plate. Horseradish peroxidase-conjugated secondary antibody (1∶2,000 in blocking solution) was added after washing the plate twice with washing buffer. The bound secondary antibody was detected using 3,3′,5,5′-tetramethylbenzidine (TMB) and stopped with 2 M H_2_SO_4_. The absorbance of the generated color was measured in an ELISA plate reader at 450 nm.

Binding of vitronectin to Gpm1 was also characterized in the presence of heparin or NaCl. Concentration-dependent binding of vitronectin to Gpm1 was measured using the method described above with various concentrations of vitronectin (0, 0.25, 0.5 and 1.0 µg). The effect of heparin was measured by pre-incubating vitronectin with increasing amounts of heparin (0, 0.01, 0.1 and 1.0 µg) for 30 min at 37°C. The same procedure was used to determine whether the binding was affected by ionic strength with the addition of various concentrations of NaCl (0, 50, 100, 150, 300 and 600 mM).

### Detection of the presence of vitronectin on human cells

To determine whether vitronectin was present on the surface of HUVEC and keratinocytes, immunological detection of vitronectin was measured using flow cytometry and laser scanning microscopy as described above with the exception of the addition of Gpm1. To detect vitronectin, rabbit polyclonal vitronectin antiserum was used as primary antibodies and Alexa 488-labeled goat anti-rabbit IgG was used as a secondary layer.

### Colocalization of Gpm1 and vitronectin on the surface of human cells

To observe the colocalization of Gpm1 with vitronectin on the surface of HUVEC and keratinocytes, laser scanning microscopy was used. Gpm1 and vitronectin were detected on the surface of human cells by following the procedure described above with the use of a monoclonal Gpm1 mouse antibody and polyclonal vitronectin rabbit antiserum. DAPI (4′,6-diamidino-2-phenylindole) was also added in the antibody mixture to visualize the nucleus of the human cells.

## Results

### Candida albicans Gpm1 binds to endothelial cells and to keratinocytes

**Figure 1:**
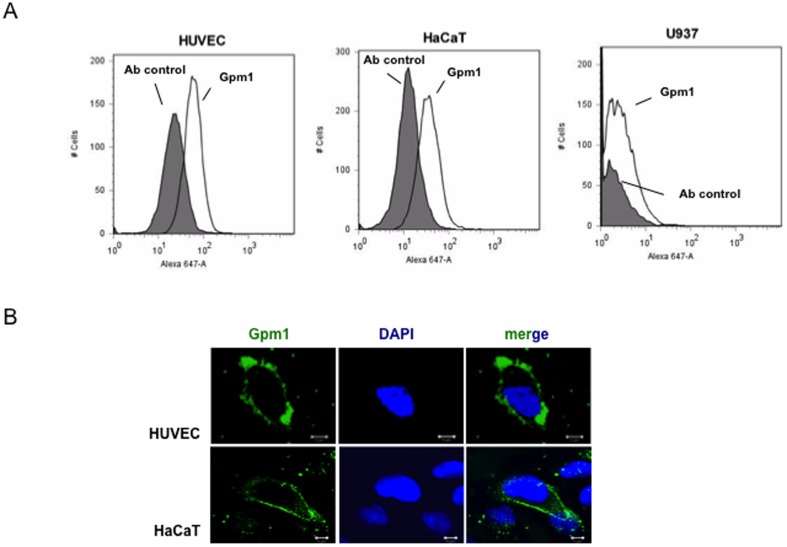
*Candida albicans* Gpm1 binds to human cells. (A) Candida Gpm1 binds to HUVEC and HaCaT. Recombinant Gpm1 was added to human endothelial cells (HUVEC), keratinocytes (HaCaT) or monocytic U937 cells. After incubation and subsequent washing, bound Gpm1 was detected in flow cytometry using rabbit Gpm1 antiserum followed by Alexa Fluor 647 goat anti-rabbit IgG. Human cells without Gpm1 were used as control. Histograms are representative of three independent experiments. (B) Binding of Gpm1 to HUVEC and HaCaT was confirmed by confocal microscopy. HUVEC (top panels) or HaCaT cells (bottom panels) were incubated with Gpm1, fixed with parafolmaldehyde and after washing, bound Gpm1 was detected with rabbit Gpm1 antiserum followed by Alexa Fluor 488 goat anti-rabbit IgG (green). The cellular DNA was stained with DAPI (blue). Scale bar = 10 µm.

*C. albicans* Gpm1 is a fungal moonlighting protein that binds the human plasma proteins Factor H, FHL-1 and plasminogen [13]. The exposure at the fungal surface together with the multifunctional role of Gpm1 in fungal immune evasion proteins suggested that Gpm1 controls additional steps of host innate immunity. To this end, first binding of Gpm1 to HUVEC, to keratinocytes (HaCaT), and to the pre-monocytic cell line U937 was analyzed. Gpm1 was bound to the cells and after washing, bound Gpm1 was detected with a specific antiserum by flow cytometry. Gpm1 bound to HUVEC (mean fluorescence intensity; MFI = 1515) and to HaCaT (MFI = 768), but did not bind to U937 cells (MFI = 54) (Figure 1A).

In addition, Gpm1 surface binding was visualized by laser scanning microscopy (LSM). Gpm1 (green fluorescence) bound to the outer surface of both HUVEC and HaCaT cells and in addition formed clusters at the cell surface (Figure 1B). Thus, *C. albicans* Gpm1 binds to human endothelial cells and to keratinocytes, but not to human monocytic U937 cells.

### *C. albicans gpm1Δ/Δ* knock-out mutant adhered with low efficiency to human endothelial cells

**Figure 2:**
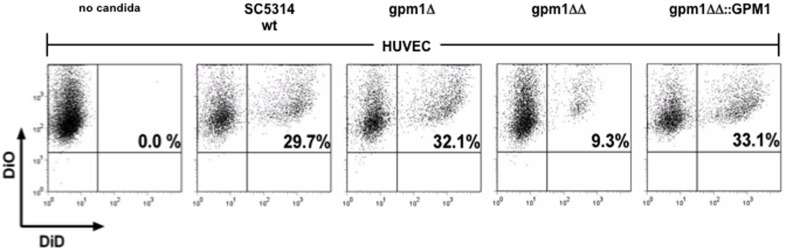
The candida *GPM1* deletion mutant bins with low intensity to human endothelial cells (HUVEC). The candida *gpm1Δ/Δ* knock-out mutant bound with low intensity to HUVEC. Yeast cells of *C. albicans* SC5314 (wild type), *gpm1Δ*, *gpm1Δ/Δ* or *gpm1Δ/Δ*::*GPM1* were labeled with DiD and incubated with DiO-labeled HUVEC for 120 min at 37°C at 5% CO_2_. After washing and detachment, HUVEC with associated (adherent and/or endocytosed) *C. albicans* cells were identified in flow cytometry as double-positive cells (DiO+, DiD+) and by the change in side scatter. HUVEC alone were detected as single-positive cells (DiO+, DiD−) were used as control.

The *C. albicans gpm1Δ/Δ* knock out mutant was used to define the role of Gpm1 in fungal adherence to HUVEC cells. The *C. albicans gpm1Δ/Δ* knock out mutant, a *gpm1Δ* heterozygous mutant, and also the reconstituted knock out strain (*gpm1Δ/Δ::GPM1*) and also the wild type candida, strain SC5314 were all labeled with the fluorescent dye DiD, and HUVEC cells with DiO HUVEC. Candida cells were identified as single positive cells (DiD^+^, DiO^−^,) and HUVEC cells alone as single-positive cells (DiD^−^, DiO^+^)(Figure 2, control). Following coincubation and after washing, adherent and ingested fungal cells were quantified by flow cytometry and HUVEC with attached *C. albicans* yeast cells were recorded as double-positive cells (DiD^+^, DiO^+^). Using wild type yeast cells, 29.7% of the HUVEC had adherent or endocytosed candida cells (Figure 2, panel II), but with the *gpm1Δ/Δ* knock-out mutant only 9.3% of the fungal cells interacted with HUVEC cells (Figure 2, panel IV). The heterozygous mutant (*gpm1Δ*) and the reconstituted strain (*gpm1Δ/Δ::GPM1*) associated with HUVEC at comparable intensities as the wild type (Figure 2, panels III and V). Thus, Gpm1 mediates *C. albicans* adherence to human endothelial cells.

### Gpm1-coated latex beads bind to human endothelial cells

**Figure 3:**
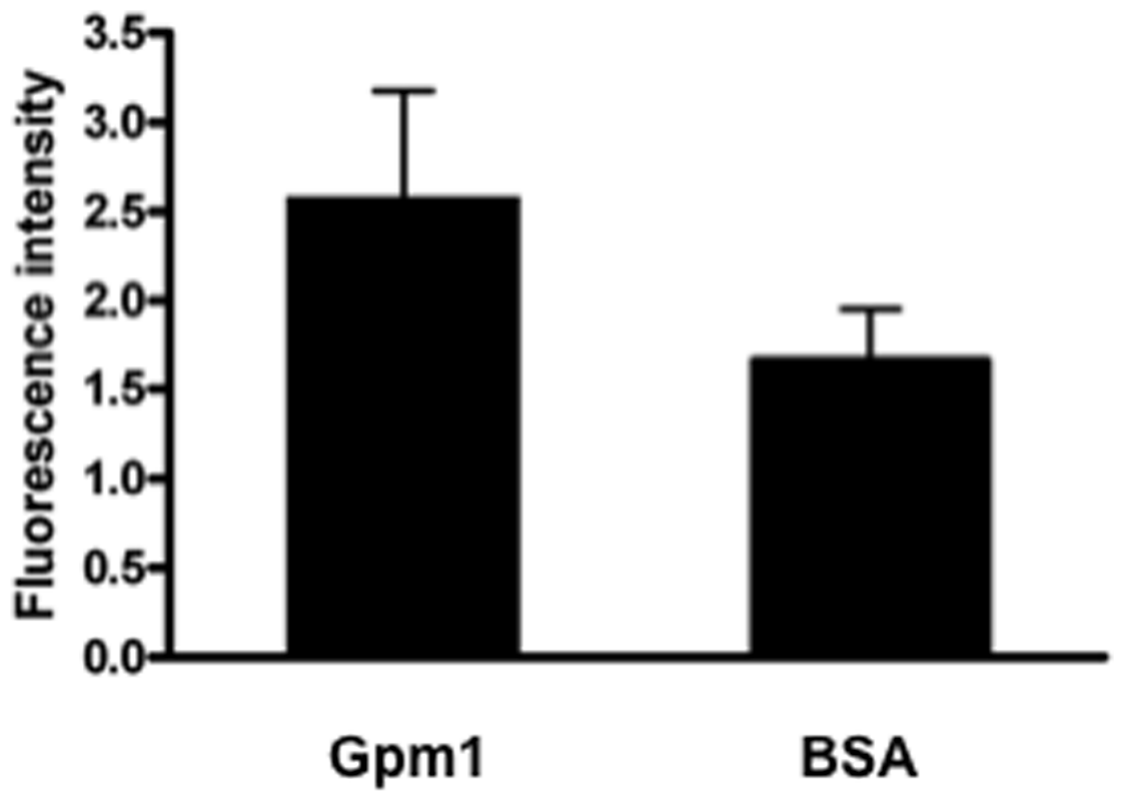
Gpm1 coated latex beads mediates adhesion to endothelial cells. Gpm1 was coated to the surface of fluorescent latex beads and the coated beads were attached to HUVEC for 45°C with 5% CO_2_. Following incubation the fluorescent latex beads were recorded and quantified by LSM. The blue fluorescence was measured per µm^2^ under the oil immersion objective lens using the ZEN 2009 software. Data are mean ± SD (error bars) of three experiments. BSA coated latex beads wer used in addition.

To define directly that Gpm1 mediates contact with human endothelial cells and to exclude an effect of other fungal surface proteins, we Gpm1 was coated to the surface of fluorescent latex bead and adherence of such Gpm1-coated latex beads to HUVEC was followed. Upon incubation and washing, the attached Gpm1-coated latex beads were identified and quantified by LSM. Gpm1-coated latex beads bound to HUVEC (MFI/µm^2^ = 2.5) and BSA-coated beads bound with lower efficiency (MFI/µm^2^ = 1.6) (Figure 3). Thus, fungal Gpm1 binds and attaches to human endothelial cells.

### Gpm1 binds the human extracellular matrix protein vitronectin

**Figure 4:**
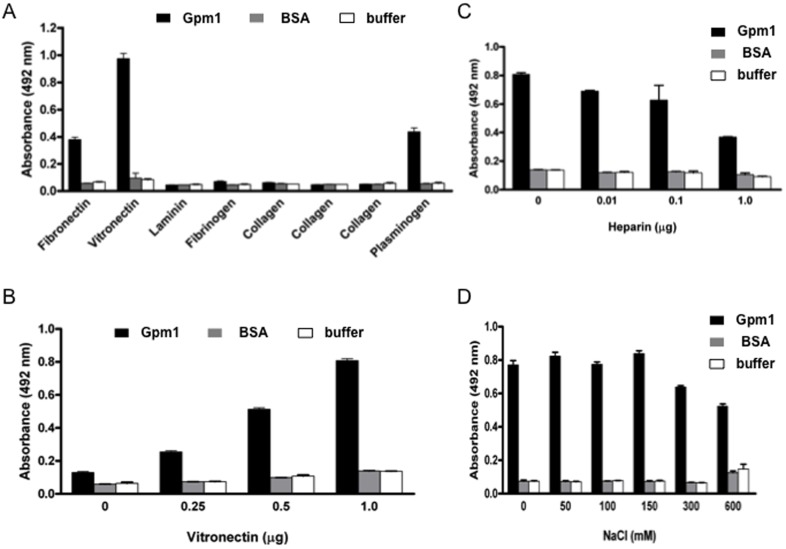
Gpm1 binds to human vitronectin. (A) Vitronectin and fibronectin bound to immobilized Gpm1. Binding of extracellular matrix (ECM) proteins to Gpm1 was determined by ELISA. Gpm1 was immobilized on a microtiter plate and fibronectin, vitronectin, laminin, fibrinogen, collagen I, collagen III, or collagen IV were added. Following washing the bound ligands were detected with antiserum specific to each ECM protein together with HRP-conjugated specific antiserum. Binding of plasminogen to Gpm1 was used as control. (B) Vitronectin dose-dependently bound to immobilized Gpm1. Gpm1 was immobilized onto a microtiter plate, vitronectin was added at the indicated amounts, and following washing bound vitronectin was detected by rabbit vitronectin antiserum, followed by HRP-conjugated polyclonal goat anti-rabbit IgG. (C) Heparin inhibited vitronectin binding to Gpm1. Vitronectin and heparin (at the indicated amounts) were pre-incubated and then added to immobilized Gpm1. Following washing bound vitronectin was detected as in (B). (D) Interaction of vitronectin with Gpm1 is affected by ionic strength. Vitronectin was pre-incubated with NaCl (at the indicated final concentrations) and the mixture was added to immobilized Gpm1. Following washing bound vitronectin was detected as in (B). BSA and buffer were used as controls. Data represent mean values ± SD (error bars) of three independent experiments.

Fungal pathogens, similar to other microbial pathogens attach to human cells often via extracellular matrix components, that are exposed at the surface of the host cells. We therefore analyzed binding of a panel of human ECM proteins to immobilized Gpm1. In this set up, fibronectin and vitronectin bound to Gpm1, but laminin, fibrinogen, collagen I, collagen III and collagen IV did not bind (Figure 4A).

Vitronectin bound with high intensity to Gpm1, and therefore the vitronectin Gpm1 interaction was analyzed in more detail. First, dose-dependent binding of vitronectin to immobilized Gpm1 was analyzed. Vitronectin bound to Gpm1, and binding was dose-dependent (Figure 4B).

Next the role of heparin on the Gpm1 vitronectin interaction was analyzed. To this end, heparin was first added to vitronectin, then the vitronectin-heparin mixture was added to immobilized Gpm1 and after washing, bound vitronectin was detected. Heparin inhibited vitronectin binding to immobilized Gpm1. The effect was dose-dependent, heparin at 0.1 µg reduced binding by approximately 20%, and at 1.0 µg heparin inhibited vitronectin binding to Gpm1 by almost 60% (Figure 4C).

In addition, the effect of ionic strength was assessed. NaCl, used at the physiological concentration of 150 mM did not affect vitronectin binding. However, at higher levels, of 300 and 600 mM NaCl, reduced vitronectin binding to Gpm1 by 24% and 38%, respectively (Figure 4D). Taken together, the vitronectin - Gpm1 interaction is affected by heparin and is also partly ionic strength dependent.

### Vitronectin blocks Gpm1 binding to human cells

**Figure 5:**
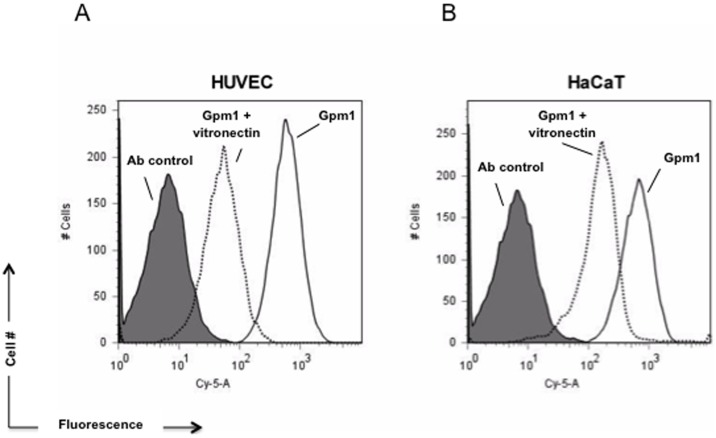
Vitronectin blocks binding of Gpm1 to human cells. Vitronectin when combined with Gpm1 decreased binding to human cells. Biotinylated Gpm1 (10 µg) was pre-incubated with vitronectin (10 µg) and then the mixture was added to HUVEC (A) or HaCaT (B). After washing, bound Gpm1 was detected using streptavidin-conjugated Cy5 by flow cytometry. Vitronectin when bound to Gpm1 (black dashed line) reduced Gpm1 binding to both human cell lines. Gpm1 binding in the absence of vitronectin shows prominent binding (black solid line). Human cells without Gpm1 and/or vitronectin was used as control (gray solid line). The data represent a representative experiment out of four independent experiments.

Gpm1 binds to human vitronectin and also to human endothelial cells and to keratinocytes. We therefore hypothesized that vitronectin exposed at the surface of these human cells represents a ligand for fungal Gpm1. To this end we assayed whether vitronectin influences Gpm1-binding to HUVEC and to HaCaT cells. Vitronectin was first combined with Gpm1 and then this mixture was added to HUVEC. After washing, bound Gpm1 was identified with a Gpm1 reacting antiserum by flow cytometry. Upon preincubation with vitronectin, Gpm1 binding to HUVEC was reduced by 91%; (vitronectin+Gpm1: MFI 1,319 vs Gpm1: MFI 15,517 (Figure 5A). Similarly, vitronectin inhibited Gpm1-binding to keratinocytes by 75% (vitronectin+Gpm1: MFI 4,096; Gpm1: MFI 16,433). Thus, vitronectin complexes fungal Gpm1 in solution and in consequence blocks Gpm1 binding to HUVEC cells.

### Vitronectin is present on the surface of human endothelial cells and keratinocytes

**Figure 6:**
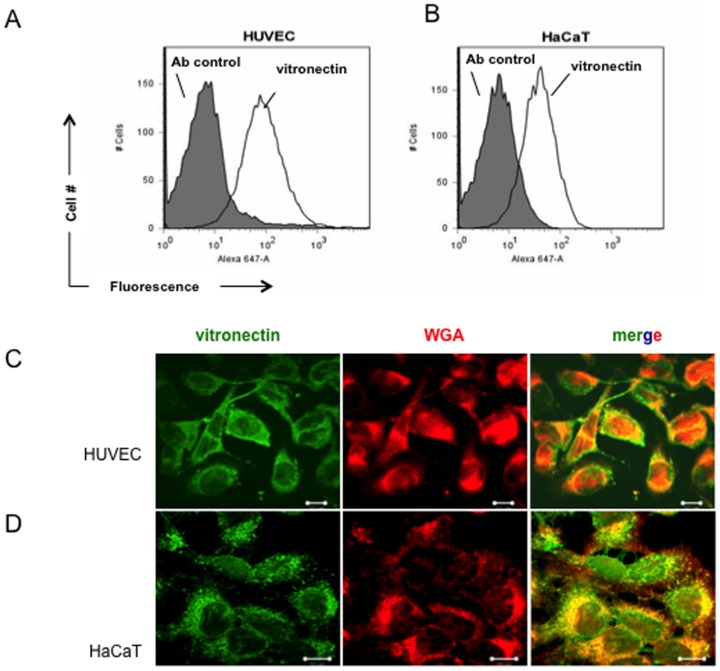
Vitronectin is present on the surface of HUVEC and HaCaT cells. Vitronectin was detected on the surface of human cells. HUVEC as well as HaCaT cells were cultivated in DMEM or RPMI, until reaching confluence. After washing, the human cells were kept in serum free medium for 24(A) Human cells were detached from the surface and vitronectin expressed on the surface of the cells was detected with specific rabbit antiserum followed by Alexa Fluor 647-conjugated goat anti-rabbit IgG as secondary antiserum by flow cytometry (solid line). The dashed line shows the same human cells that were treated with the secondary antiserum as a control. (B) Expression of vitronectin by human cells was visualized by confocal microscopy. Human cells were grown on coverslip in 24-well plate with appropriate medium until reaching confluence. After washing, the cells were maintained in serum-free medium for 24 h. Then the cells were fixed with paraformaldehyde (3%) and after extensive washing, vitronectin present on the cell surface was detected by rabbit vitronectin antiserum followed by Alexa Fluor 488-conjugated goat anti-rabbit IgG (green). The membrane of the human cells was visualized with Texas red-conjugated wheat germ agglutinin (red). Scale bar = 20 µm. The data represent a representative experiment out of four independent experiments.

As vitronectin binds to *C. albicans* Gpm1 and blocks Gpm1-binding to human endothelial cells, we hypothesized that vitronectin, present at the surface of human cells serves as a cell ligand for Gpm1. Therefore, we analyzed whether vitronectin is exposed at the surface of human endothelial cells and keratinocytes, that were cultivated in serum-free medium. In this case vitronectin was detected on the surface of both HUVEC (MFI = 2,153) and HaCaT cells (MFI = 819). (Figure 6)

In addition vitronectin was detected at the surface of human keratinocytes by confocal microscopy (Figure 6B, bottom panel). Vitronectin (green) was exposed at the outside, at the cell membrane, which was labeled with wheat germ agglutinin (red). Vitronectin and the membrane constituents colocalized at the cell surface, as revealed by the yellow signal, upon merging the two images (Figure 6B, right upper panel). Taken together, Vitronectin is present on the surface of both HUVEC and HaCaT.

### Gpm1 and Vitronectin colocalize at the surface of human cells

**Figure 7:**
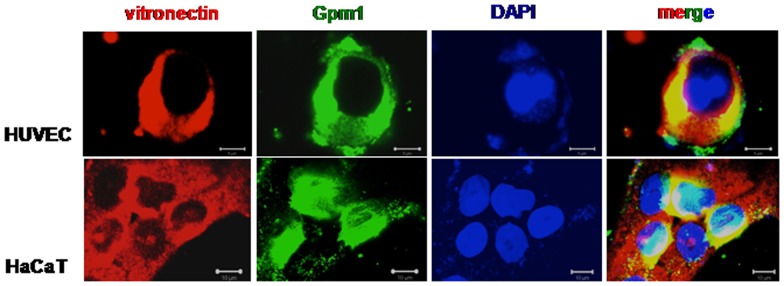
Gpm1 and vitronectin colocalize at the surface of human cells. Vitronectin (red) and Gpm1 (green) colocalizes at the surface of human cells. Recombinant Gpm1 was attached to HUVEC or HaCaT cells that were kept in serum-free medium for 24 h. Following washing the cells were fixed with paraformaldehyde (3%). Gpm1 was detected with a monoclonal mouse Gpm1 antibody followed by Alexa Fluor 488-conjugated goat anti-mouse IgG. Vitronectin expressed on the surface of the cells was detected by polyclonal rabbit vitronectin antiserum followed by Alexa Fluor 647-conjugated goat anti-rabbit IgG. DNA of human cells was stained with DAPI. Scale bar = 10 µm. The images show a representative experiment out of four independent experiments.

To prove that Gpm1 binds via vitronectin to the surface of human cells, Gpm1 ws bound to the surface of HUVEC cells and keratinocytes, HaCaT which were cultivated in serum-free medium. Following washing vitronectin (red) and Gpm1 (green) were localized by immune fluorescence (Figure 7, left panel and middle left panel). A merge of the images showed co-localization of gpm1 and vitronectin at the cell surface (yellow signal) (Figure 7, right panels). Thus, the fungal Gpm1 protein binds to human vitronectin, which is exposed on the surface of human endothelial cells and keratinocytes.

## Discussion

*C. albicans* virulence is mediated by complement and innate immune escape and also by binding and adhesion of the pathogenic yeast to human endothelial cells, as well as keratinocytes. Attachment and interaction with host endothelial cells and keratinocytes is central for fugal tissue dissemination. To understand how the fungal pathogen *C. albicans* attaches to and invades human cells, we aimed to identify fungal surface proteins that bind to human endothelial cells and to keratinocytes. In addition we were interested to identify the corresponding surface ligands that exposed on the human cells. Here, we identify Gpm1, the candida surface protein as a fungal adhesion protein, that binds to human endothelial cells and to keratinocytes. In addition, vitronectin was identified as a new Gpm1 interacting protein. Vitronectin exposed at the surface of human endothelial cells and keratinocytes serves as a ligand for candida Gpm1.

*C. albicans* is one of the leading fungal pathogens that cause cutaneous infections. *C. albicans* adheres to human endothelial cells, that line the blood vessel via specific surface proteins. Thus upon skin infection, Gpm1 assists in fungal invasion of epidermal layers. *C. albicans* uses several surface proteins to contact human endothelial cells. In addition to Gpm1 candida also uses Als3 and Ssa1 for interaction with human endothelial cells [47], [48], [49]. Thus candida apparently uses a combination of fungal adhesions proteins that aid in attachment, and in combination with acquired and endogenous proteases aid in destruction of endothelial barriers [50], [51], [52]. Gpm1, also mannose and phospholipomannan moieties, central fungal cell wall components contribute to fungal attachment to human keratinocytes [33], [53], [54]. *C. albicans* binds to human keratinocytes and here we identify Gpm1 as the first fungal surface protein that mediates attachment of human keratinocytes. In addition to

Here we identify candida Gpm1, as the fungal surface protein that binds the human adhesive glycoprotein vitronectin. Gpm1, as a fungal adhesin, contributes to complement and immune escape, cell adhesion, invasion and colonization. Human vitronectin, bound to the candida surface contributes to fungal adherence to human endothelial cells and to keratinocytes. Candida Gpm1 binds to vitronectin exposed on the surface of human endothelial cells and keratinocytes, but not to monocytic U937 cells. Vitronectin which is exposed at high levels at the surface of human endothelial cells and keratinocytes and Gpm1 co-localizes. Heparin influenced the Gpm1-vitronectin interaction, thus indicating that the heparin-binding regions of vitronectin are involved in this interaction.

Candia apparently expresses several vitronectin-binding proteins at the fungal surface. In addition to Gpm1, two fungal vitronectin binding surface proteins were identified, as the two integrin-like receptors that cross-react with antibodies against human integrins α_v_β_3_ and α_v_β_5_ and that have an apparent masses of 84–130 kDa [29], [30]. However, Gpm1 is distinct from these two proteins, Gpm1 has a lower molecular mass and a different immune reactivity. Recombinant Gpm1 did not react with monoclonal antibody specific for the human integrin receptor α_v_β_3_ (data not shown). Thus *C. albicans* has at least three vitronectin-binding proteins. Using radiolabelled vitronectin for ligand blotting, a fungal 30-kDa surface protein was previously identified as a vitronectin ligand [45]. These authors also showed that vitronectin binds to candida yeast cells via the glycosaminoglycan-binding regions. In this study the fungal vitronectin binding protein was neither purified, identified nor cloned. However the similar binding profiles, the role of the heparin-binding domains and the almost identical masses of Gpm1 with the described protein suggest that the 30 kDa Gpm1 protein actually represents the fungal surface protein described by these authors.

Gpm1 is a multifunctional moonlighting protein [13], [55]. Gpm1 is a moonlighting protein that is present at the fungal surface and as a cytoplasmic protein regulates glycolysis. Cytoplasmic Gpm1 converts 3-phosphoglycerate to 2-phosphoglycerate and the reverse reaction during gluconeogenesis. As a surface protein, Gpm1 is expressed on *C. albicans* yeast cells and also on hyphae, particularly at the tip of hyphae [13]. At the fungal surface, Gpm1 binds several human plasma proteins, including vitronectin and the complement regulators Factor H, FHL-1, plasminogen and high molecular weight kininogen, the precursor of kinin [13], [53], [54]. Microbial Moonlighting proteins in general lack a typical secretion signals that direct transport to the cell surface via the secretory pathway and currently it is not understood how these proteins are transported to the cell surface (55). *C. albicans* expresses additional moonlighting proteins that are surface-exposed and that are also present both in the cytoplasma. These candida moonlighting proteins, which include, enolase (Eno1), glyceraldehyde-3-phosphate dehydrogenase (GAPDH) and glycerol-3-phosphate dehydrogenase 2 (Gpd2) each of which binds a series of human plasma proteins and regulates multiple steps in fungal virulence, including immune evasion, immune defense, cell adhesion and, as well ECM interaction [56], [57], [58].

Gpm1, is a fungal vitronectin, Factor H and plasminogen-binding immune evasion protein. At present, a three candida vitronectin-, eleven plasminogen- and four Factor H-binding proteins are identified [6], [23], [24]. Each fungal surface protein represents a virulence factor that contribute to fungal immune camouflaging and that acts as adhesins (6, 7, 10). This large number of fungal immune escape proteins shows that *C. albicans* uses an array of surface proteins for immune escape. One single fungal immune escape proteins acquires a whole panel of soluble host immune- and coagulation regulators and binds several ECM components. With such a broad binding repertoire a single fungal escape protein controls multiple steps in fungal immune escape. Such multivalent immune escape strategies provide important insights into fungal immune escape and attachment to host cells and ECM components.

Thus Gpm1 is a new candida vitronectin-binding protein which mediates multiple steps in fungal immune evasion and virulence. Deepening the understanding how fungi interact with the human host. Targeting candida Gpm1 and blocking the Gpm1 vitronectin interaction may lead to a directed therapeutic approach to modulate or even block fungal infection.
